# Differential response to heat stress in outer and inner onion bulb scales

**DOI:** 10.1093/jxb/ery189

**Published:** 2018-05-18

**Authors:** Ortal Galsurker, Adi Doron-Faigenboim, Paula Teper-Bamnolker, Avinoam Daus, Amnon Lers, Dani Eshel

**Affiliations:** 1 Department of Postharvest Science of Fresh Produce, The Volcani Center, Agricultural Research Organization, Rishon LeZion, Israel; 2 The Robert H. Smith Institute of Field Crops and Vegetables, The Robert H. Smith Faculty of Agriculture, Food and Environment, The Hebrew University of Jerusalem, Rehovot, Israel; 3 Institute of Plant Sciences, The Volcani Center, Agricultural Research Organization, Rishon LeZion, Israel

**Keywords:** Curing, defense response, heat tolerance, heat treatment, onion scale, oxidation, tissue browning, transcriptome

## Abstract

The formation of brown protective skin in onion bulbs can be induced by rapid post-harvest heat treatment. Onions that are peeled to different depths and are exposed to heat stress show that only the outer scales form the dry brown skin, whereas the inner scales maintain high water content and do not change color. Our study demonstrates that browning of the outer scale during heat treatment is due to an enzymatic process that is associated with high levels of oxidation components, such as peroxidase and quercetin glucoside. *De novo* transcriptome analysis revealed differential molecular responses of the outer and inner scales to heat stress. Genes involved in lipid metabolism, oxidation pathways, and cell-wall modification were highly expressed in the outer scale during heating. Defense response-related genes such as those encoding heat-shock proteins, antioxidative stress defense, or production of osmoprotectant metabolites were mostly induced in the inner scale in response to heat exposure. These transcriptomic data led to a conceptual model that suggests sequential processes for the development of browning and desiccation of the outer scale versus processes associated with defense response and heat tolerance in the inner scales.

## Teaser

Differential gene expression in the outer and inner scales of onion bulbs in response to heat stress accounts for the fact that only outer scales can form a protective dry brown skin.

## Introduction

Onion bulbs (*Allium cepa* L.) are one of the most economically important *Allium* crops and are highly appreciated by consumers because of their distinctive sensory and beneficial compounds (Mota *et al.*, 2010). The bulb morphology is composed of scales ordered according to their chronological age from inner, younger, to outer, older, scales (Galsurker *et al.*, 2017). A typical onion bulb at maturity has one-to-three dry skins originating from scales that enclose sequential thin outer scales, which in turn enclose several swollen, inner fleshy scales (Brewster, 2008; Galsurker *et al.*, 2017). Onion-skin formation involves activation of a programmed cell death (PCD) mechanism together with desiccation and development of browning in the outer scales; meanwhile, in parallel, metabolism-maintenance processes occur in the inner, younger, fleshy scales (Galsurker *et al.*, 2017). As the outer and inner scales differ in function and physiological age, they can serve as an excellent system to compare their responses to abiotic stress.

Bulb curing is an important post-harvest heat treatment that is commonly used to dry out and harden the necks and outermost scales of the bulbs, thus providing completely dry, brown protective skins around the bulb (Maw *et al.*, 2004). These skins provide bulb defense and minimize yield losses due to water loss and pathogen infection, resulting in increased storage potential and maintenance of bulb quality during storage (Maude *et al.*, 1984; Chope *et al.*, 2012). Bulbs can be cured either in the field, by allowing them to dry in the sun, or by artificial curing in storage, during which temperature and its rate of change, humidity, and curing duration can be controlled.

Several studies have focused on the effects of different curing methods on post-harvest bulb quality during storage (Nega *et al.*, 2015). Curing temperature and duration can affect bulb quality and yield losses, as they are closely related to the frequency of storage diseases (Schroeder *et al.*, 2012; Bansal *et al.*, 2015). Other studies have explored methodological improvements and modifications of the curing process by reducing the temperature and duration of the curing and drying periods, thereby conserving energy (Chope and Terry, 2010). Eshel *et al.* (2014) developed a fast-curing method by applying a rapid and controlled heat treatment at 98% relative humidity (RH) and 30 °C for 9 d, which improved onion bulb quality due to an increase in the number of outer dry skins, and in their physical strength. Downes *et al.* (2009) found that different curing temperatures affect the biochemical composition of the skin, mainly with regards to flavonols and anthocyanins and their contribution to the color change of skins during curing.

While most studies have mainly described technological and methodological aspects of curing methods, the biological processes involved with curing are not clear. The present study provides a comprehensive description of the differential gene-expression patterns in outer and inner scales during post-harvest curing. We examined the consequences of a heat-curing treatment (33 °C and 98% RH) on individual outer and inner scales, and the differential effects on their morphological, biochemical, and transcriptional characteristics. We propose that the differential transcriptomes observed between outer and inner scales in response to the heat treatment may be responsible for the different effects of the curing treatment on their morphological and structural features.

## Materials and methods

### Plant material

A commercial brown onion cultivar, Orlando, was grown in sandy soil in the north-western Negev desert in the years 2014–2016. The onions were not sprayed with maleic hydrazide before leaf drop, as per common agricultural practice, and did not undergo field curing. Onions were harvested manually at 80–100% fallen leaves (top-down) and the leaves were removed with a sharp knife, leaving a ~10-cm long neck above the bulb, as described previously (Eshel *et al.*, 2014). The onions were placed in a dark storage room at 2 °C and 70% RH for 4–8 weeks. Experiments were conducted with undamaged bulbs of standard shape. Bulbs freshly harvested from the field contain a single, completely dry skin (‘skin’) and, underneath it, several thin yellowish scales. These yellowish scales, numbered 1–4, have the ability to develop into additional dry skins over the course of a few days during fast-curing (FC) (Galsurker *et al.*, 2017). In several experiments, muddy skin was removed to expose the 1st scale, and bulbs were separated into different successive scales, which were numbered from the exterior to interior of the bulb as described previously by Galsurker *et al.* (2017).

Powdered scale material was prepared by freezing the individual scales in liquid nitrogen and grinding to a fine powder using a liquid nitrogen grinder/mill (IKA, Germany). The freeze-dried powdered scales were kept at –20 °C until use.

### Heat treatment

Heat treatment was performed as described in the Introduction for FC (Eshel *et al.*, 2014) with minor modifications. About 60 onion bulbs were peeled to three different depths by (i) removing the skin to expose the 1st scale, (ii) removing the skin and the next four scales to expose the 5th scale from the outside, or (iii) removing the skin and the next seven scales, to expose the 8th scale. Peeled bulbs were subjected to heat treatment by incubating at 33 °C under either low or high RH (45% or 98%, respectively), created by an ultrasonic fogger (SMD Technology, Rehovot, Israel), for up to 22 d.

For the detached-scale experiments, bulbs were peeled to remove the dry skin and expose the 1st scale, and then scales were separated and numbered from the outside to inside (Galsurker *et al.*, 2017). Detached scales were incubated at 33 °C under 98% RH in an incubator (Binder KBF720, Binder Instrument Co., Germany) and sampled over time.

### Color-development and water-loss measurements

Color development and water loss in the peeled onions were measured after 22 d of heat treatment (33 °C at 45% or 98% RH). Scale color was measured by diffuse reflectance with a Minolta CR-300 Chroma Meter (Konica Minolta Sensing, Osaka, Japan) and expressed as the hue angle (*H*, in degrees). Measurements were performed on each onion bulb around its widest diameter. Water loss from the exposed scale was determined by measuring the relative water content (RWC) following the heat treatment. For the water-loss measurement, the exposed scales were detached from the bulbs, six 2-cm diameter discs were punched from each scale with a cork borer and weighed for initial fresh weight (FW). After weighing, the discs were soaked in distilled water for 24 h at room temperature, and then carefully blotted dry with tissue paper to determine saturated weight (SW). The discs were then dried in an oven at 70 °C for at least 24 h to measure the dry weight (DW). RWC was calculated as follows:

$$\text{RWC }\left(\%\right)=\frac{\left(FW-DW\right)\times 100}{\left(\text{SW}-\text{DW}\right)}$$ (1)

### *In vitro* browning of powdered scales

Color measurements of the powders from the 1st and 5th scales were determined using an *in vitro* assay after heat incubation. Samples of 0.4 g of powder were suspended in 2 ml of 0.03 M phosphate/citrate buffer solution at either pH 4 or pH 7. The sample suspensions were heat-incubated in a water bath at 33 °C for 72 h, and color was measured by diffuse reflectance with a Minolta CR-300 Chroma Meter and expressed as values of *H*. The suspension of powder from the 1st scale was boiled for 10 min at 100 °C and incubated at 33 °C for 72 h, then color was measured in the same manner. In another experiment, powder suspensions of the 1st scale were adjusted to pH values ranging from 3.5 to 8, incubated at 33 °C for 72 h, and then color was measured to determine the optimum pH of the buffer suspension for browning.

### Quercetin glucoside (QG) content

Detached scales (1st, 3rd, and 5th) were sampled 0, 2, and 4 d after heat treatment, freeze-dried, and ground as descried previously (Galsurker *et al.*, 2017). Powdered samples from 0.1 g of dry matter were extracted with 3 ml of 80% ethanol by vortexing for 3 min in 15-ml tubes. The homogenate was then centrifuged at 10000 *g* for 5 min. The supernatant was transferred to a new tube, and this process was repeated twice with the pellet residue. The supernatants were pooled, filtered through Whatman No. 1 filter paper and stored at –20 °C until analysis. The ethanol extracts were thawed, vortexed, and diluted 10:1 with 80% ethanol. The QG was quantified spectrophotometrically according to Lombard *et al.* (2002) with some modifications. Absorbance readings were performed in triplicate at 362 nm using an ELISA Plate Reader spectrophotometer (Enspire 2003 Multi Label Reader, Perkin-Elmer). The results were expressed as QG concentration according to a spiraeoside (quercetin 4-glucoside) calibration curve.

### Peroxidase (POD) activity

For enzyme extraction, powdered samples from 0.1 g of dry matter were suspended in 3 ml extraction buffer (100 mM potassium phosphate, pH 7, 0.8 M NaCl, 1 mM EDTA, and 1 mM dithiothreitol, 0.3% w/v Triton X-100) under ice-cold conditions. The homogenates were centrifuged at 10000 *g* for 15 min at 4 °C, and the pellets were resuspended in the same buffer and centrifuged again. The resulting supernatants were pooled with the previous ones and used for determination of POD activity. Soluble POD was assayed in microtiter plates using 3,3’,5,5’-tetramethylbenzidine (TMB) as the substrate, made up at 20 mg ml^–1^ in DMSO and stored at –20 °C. The activity was determined by adding 5 µl of the sample extraction to 95 µl of 0.1 M sodium acetate buffer, pH 6, containing 0.1 mg ml^–1^ TMB and 0.5 µl ml^–1^ of 6% (w/v) H_2_O_2_. There was no reaction in the absence of H_2_O_2_. The absorbance was monitored at 570 nm and results were expressed as ΔA_570_OD min^−1^ mg DW^−1^.

### Ion-leakage assay

Using a 20-mm diameter cork borer, two discs each from the 1st and 5th scales were excised and placed into 50-ml plastic tubes containing 20 ml of an isotonic solution of 0.2 M mannitol. Ion leakage was measured as the change in electrical conductivity (*σ*) using a conductivity meter (CON 700 Conductivity/°C/°F Bench Meter, USA) over 4 h at 25 °C. Ion leakage was calculated as the percentage of total electrical conductivity of the sample. Total conductivity was measured from samples that were autoclaved (121 °C) and cooled to room temperature, resulting in 100% ruptured cells:

$$\text{Ion leakage }\left(\%\right)=\frac{\text{Conductivity}\left(\text{σ}=4\text{ h}\right)\times 100}{\text{Total conductivity}}$$ (2)

### Respiration rate

Samples of the 1st and 5th scales were used to measure respiration. In each case, eight individual scales were placed in 2-l glass jars and incubated at 33 °C under 98% RH for up to 8 d. For measurement of respiration rate, the jars were hermetically sealed with lids equipped with rubber septa for 1 h, and samples were withdrawn into gas-tight syringes and injected into a gas chromatograph for determination of CO_2_-production rates. Measurements were taken every 2 d during the heat treatment. The CO_2_ concentration was measured in a Shimadzu TM GC-2014 with thermal conductivity detector and Porapack Q columns. Results were expressed as mg CO_2_ g^−1^ h^−1^.

### RNA isolation, cDNA library construction, and RNA-Seq

Samples of the 1st and 5th scales were heat-treated at 33 °C under 98% RH for 0, 24, and 48 h, and were used for RNA isolation, cDNA synthesis, and sequencing (two biological replicates). Samples were frozen in liquid nitrogen and stored at –80 °C until RNA extraction. Total RNA of each sample was extracted as described by Galsurker *et al.* (2017) using the CTAB protocol (Chang *et al.*, 1993). Samples were treated with DNase (Epicentre, Madison, WI, USA) according to the manufacturer’s instructions. RNA purity and integrity were verified by a RNA 6000 Nano Assay on an Agilent 2100 BioAnalyzer with a minimum RNA integrity number value of 7. Library preparation and sequencing were performed at the Genome Center, Life Sciences and Engineering, Technion, Israel. Fourteen single-end RNA-Seq libraries with a length of 100 nucleotides were prepared using the Illumina HiSeq2000 and TrueSeq protocols.

### Transcriptome analysis

Raw reads were subjected to filtering and cleaning as follows. The SortMeRNA tool was used to filter out rRNA (Kopylova *et al.*, 2012), and then the FASTX Toolkit (http://hannonlab.cshl.edu/fastx_toolkit/, version 0.0.13.2) was used for: (i) trimming read-end nucleotides with quality scores <30 using fastq_quality_trimmer; and (ii) removing reads with less than 70% base pairs with quality score ≤30 using fastq_quality_filter. A recently released transcriptome catalog was employed as a reference (NCBI bioproject PRJNA326316; Galsurker *et al.*, 2017) as follows. A total of ~270 million cleaned reads, obtained after processing and cleaning, were assembled *de novo* using the Trinity software (version trinityrnaseq_r20140717 2.1.1; Grabherr *et al.*, 2011) with the trimmomatic option to remove adaptors (Bolger *et al.*, 2014). Filtering of the likely contig artifacts and low-expressed contigs was then applied. Sequencing data were deposited in the NCBI Sequence Read Archive (SRA) database (as Bioproject PRJNA326316). The cleaned reads from each library were aligned separately to the transcriptome catalog using Bowtie aligner (Langmead *et al.*, 2009), and the abundance estimation was calculated with expectation maximization (RSEM), which handles read-mapping uncertainty with a statistical model by estimating maximum-likelihood expression levels (Li and Dewey, 2011). Analysis of differential expression was performed using the edgeR software suite (Robinson *et al.*, 2010). Transcripts that were more than four-fold differentially expressed with a false discovery-corrected statistical significance of at most 0.001 and log_2_ of the fold-change lower than –2 or greater than 2 were considered differentially expressed (Benjamini and Hochberg, 1995). The expression patterns of the transcripts in the different samples were studied using cluster analysis of the differentially expressed transcripts in at least one pairwise sample comparison. Then, following the Trinity protocol (Haas *et al.*, 2013), expression normalization was designed using the trimmed mean of M-values (TMM), following fragments per feature kilobase per million reads mapped (FPKM) calculations. Hierarchical clustering of the normalized gene expression (using centralized and log_2_ transformation; Haas *et al.*, 2013) and heat-map visualization were performed using R Bioconductor (Gentleman *et al.*, 2004). We used the ‘Venny’ tool (http://bioinfogp.cnb.csic.es/tools/venny_old/venny.php) for construction of Venn diagrams.

### Gene ontology (GO) enrichment

GO and Kyoto Encyclopedia of Genes and Genomes (KEGG) annotations were examined using the GSEA server (http://plantgrn.noble.org/iPPServer/). GO enrichment analysis was carried out using the Blast2GO program (Conesa *et al.*, 2005) based on GO terms obtained from the onion transcriptome catalog (Galsurker *et al.*, 2017) that was annotated using BLASTx searches (*E*<10^−5^) (Altschul *et al.*, 1990) against databases of Arabidopsis (http://www.arabidopsis.org), *Oryza sativa* (www.phytozome.org), and SwissProt proteins (http://www.uniprot.org/; ftp://ftp.uniprot.org/pub/databases/uniprot/current_release/knowledgebase/complete/, file: niprot_sprot.fasta.gz). GO enrichment analysis was performed based on Fisher’s Exact Test (Upton, 1992) with multiple testing correction of false discovery rate (FDR) (Benjamini and Hochberg, 1995). The threshold was set to a FDR with corrected *P*-value of less than 0.05. GO analysis was performed by comparing the GO terms in the test sample to those in a background reference. GO provides a structured and controlled terminology to define gene products according to three domains: molecular function (the biochemical activity of a gene product), biological process (operations or sets of molecular events to which the gene product contributes), and cellular component (cell parts in which a gene product is active).

## Results

### Differential physiological responses of outer and inner scales to heat treatment

**Fig. 1.**
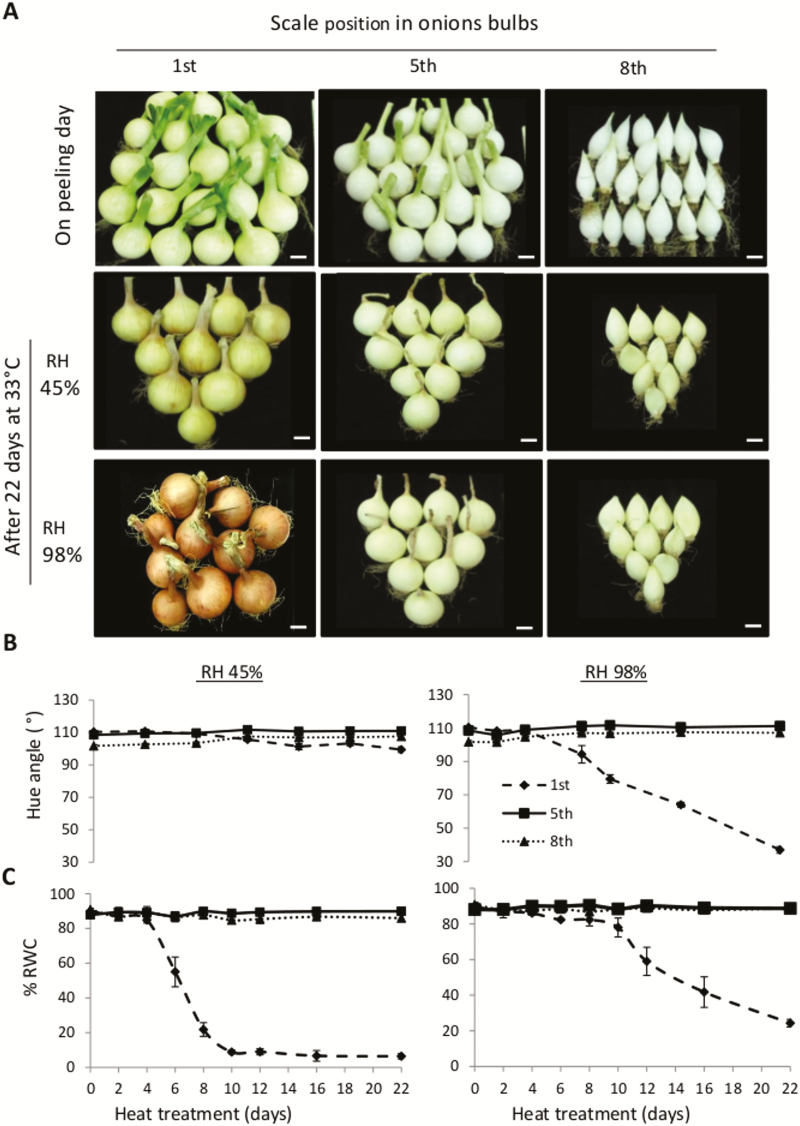
Only the outer onion scale forms dry skin during heat treatment. Onion bulbs were peeled to three depths, 1st, 5th, and 8th scales, and exposed to heat treatment at 33 °C under 45% or 98% RH. (A) Peeled onions on the day of peeling and after 22 d of heat treatment. Scale bars are 2 cm. (B) Color changes of the bulb during 22 d of heat treatment. (C) Relative water content (RWC) of the exposed scales at each peeling depth during 22 d of heat treatment. Data are means (±SE) of six replicates.

To determine whether deeper scales in the bulb are able to turn into skin and desiccate following heat treatment, onions were peeled to three depths (1st, 5th, and 8th scales). The peeled onions were heated to 33 °C under low or high humidity (45% or 98% RH, respectively) for 22 d. Color development and water loss of the peeled bulbs were measured during the treatments. Among the scales tested, only the 1st was able to form a dry brown skin following the heat treatment (Fig. 1A). High humidity (98% RH) during heating caused the formation of a dark red-brown integral skin, whereas a cracked and pale yellow-colored skin was observed at 45% RH. The *H* value of the 1st scale dramatically decreased from 110° to 37° after 22 d of heating under 98% RH, compared to a negligible decrease from 110° to 99° after heating under 45% RH (Fig. 1B). In contrast, the *H* values of the 5th and 8th scales did not change significantly over 22 d of heat treatment under either humidity level, with values remaining above 100° (Fig. 1B).

To determine water loss from the exposed scales, RWC was measured over the 22 d of heat incubation (Fig. 1C). The decrease in RWC in the 1st scale under 45% RH began earlier, after 4 d, and reached a minimum of 6%, as compared to a decrease that started after 10 d in the 98% RH treatment and reached a minimum of 24% after 22 d of heating. No significant differences in RWC were found in the 5th and 8th scales during the heat treatments under either humidity (Fig. 1C). These findings revealed that development of browning in the 1st outer scale following heat treatment required a certain level of water content in the scale and did not take place under dry (low humidity) conditions.

**Fig. 2.**
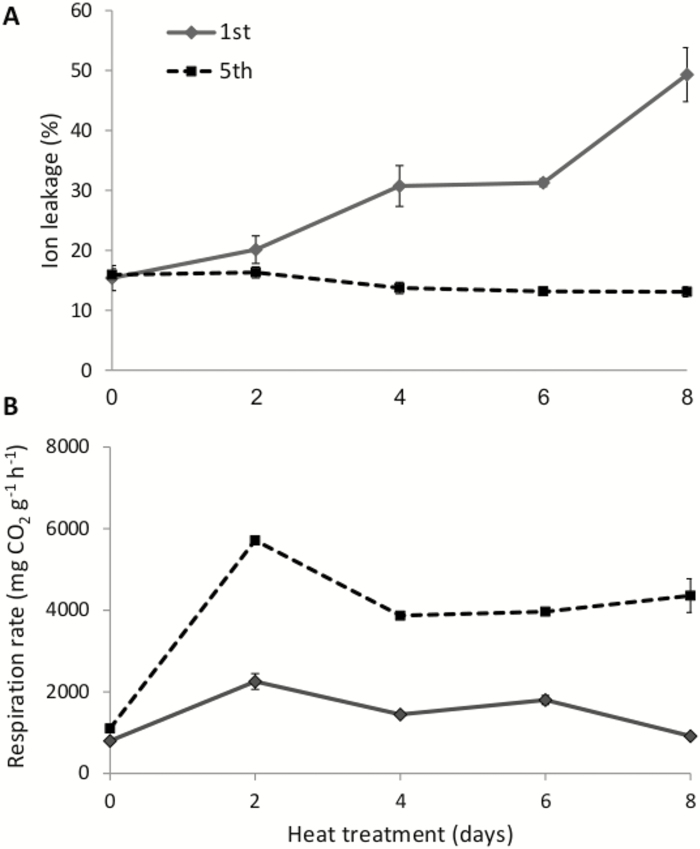
Heat treatment induces higher ion leakage from the outer scale and higher respiration rate in the inner scales of onion bulbs. The 1st and 5th scales were detached from the bulb and exposed to heat treatment at 33 °C, 98% RH. (A) Ion leakage and (B) respiration rate were measured for up to 8 d. Data are means (±SE) of six replicates.

To identify additional differences between the scales in response to heat treatment, ion leakage and respiration rate were measured in the 1st (outer) and 5th (inner) scales during heat treatment. Ion leakage in the 1st scale increased gradually from 16% to 49% after 8 d of heat treatment; in contrast, there were no significant differences in ion leakage in the 5th scale (Fig. 2A). The respiration rate in the 5th scale increased and peaked after 2 d and then decreased gradually to a plateau after 8 d of heat treatment. The respiration rate in the 1st scale was lower and did not change significantly during the heat treatment (Fig. 2B). Taken together, the results indicated that the 1st and 5th scales had different metabolic activities and responded differently to the heat stress.

### Heat-induced outer-scale browning involves oxidation processes

**Fig. 3.**
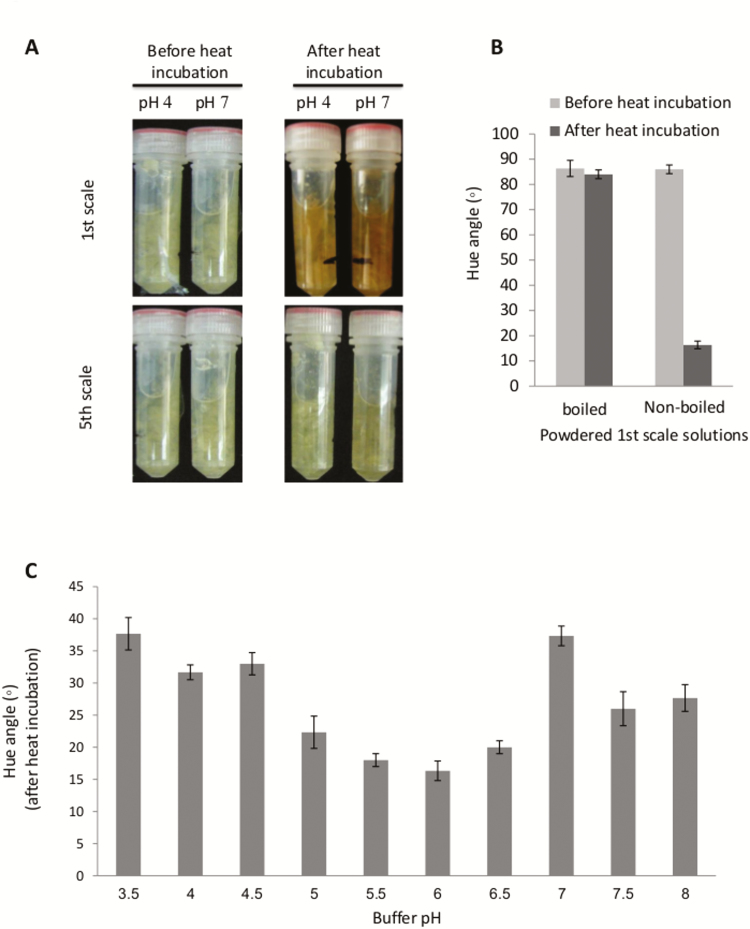
Color change of a liquid suspension of onion scale powder is induced by heat treatment only for the outer scale. (A) Powders of the 1st and 5th scales were heated for 72 h at 33 °C in 0.03 M citrate phosphate buffer at pH 4 or 7. (B) Effect of boiling on the color (described by the hue angle) of the powdered 1st scale suspension after heat incubation. (C) Effect of pH on the color of the powdered 1st scale suspension treated for 72 h at 33 °C in 0.03 M citrate phosphate, pH 3.5–8.0. Data are means (±SE) of five replicates.

We wanted to determine whether outer-scale browning following heat treatment was derived from induced enzymatic activity in conjunction with loss of cellular compartmentalization, enabling direct interaction between the enzymes and the relevant substrates. To simulate loss of cellular compartmentalization, the 1st and 5th scales were ground to fine powders, suspended in phosphate/citrate buffer solution at acidic (4) and neutral (7) pH, and incubated at 33 °C to detect color development. After 72 h of incubation, only the powder from the 1st scale changed from a yellowish color to brown, while the powder from the 5th scale remained whitish-yellow (Fig. 3A). These results indicated that the 1st, outer, scale contained internal components that could mediate scale browning, and that these components may be reduced in the inner scales.

Boiling of the powdered 1st scale suspension for 10 min followed by incubation at 33 °C for 72 h was used to assess the involvement of enzymatic activity in the browning process. Boiling prevented development of the brown color, with no significant change in the *H* value (from 86° to 84°), whereas the non-boiled control sample formed a typical brown color after the heat incubation (*H* value decreased from 86° to 32°) (Fig. 3B). Browning of the 1st scale suspension was found to occur over a pH range of 3.5– 8 and was highest at pH 6, suggesting that this is the optimum for the enzyme causing the browning (probably POD; Fig. 3C).

**Fig. 4.**
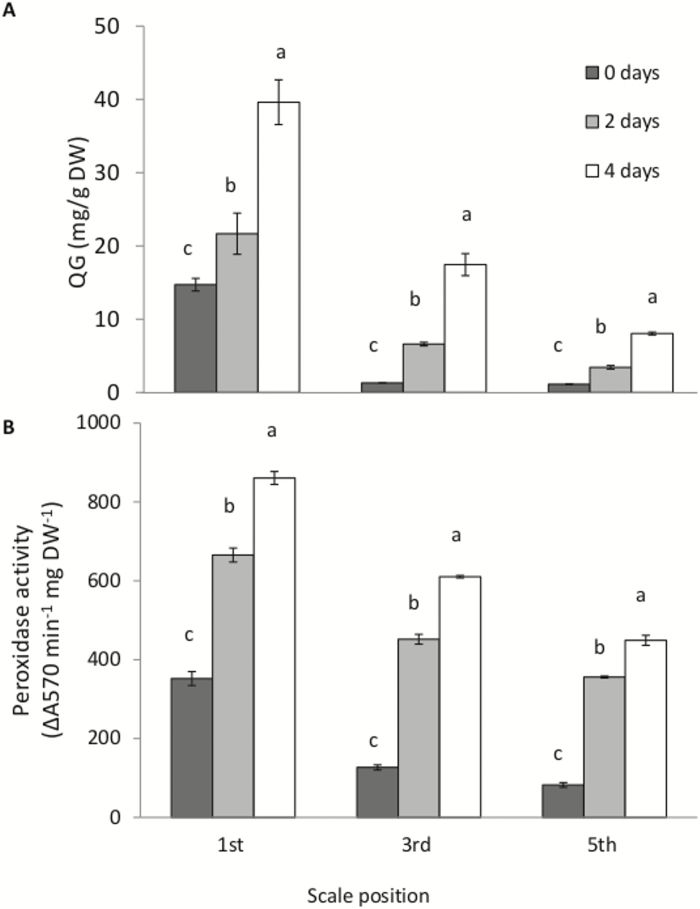
Quercetin glucoside (QG) content and peroxidase activity are higher in the outer onion scale following heat treatment. (A) QG contents and (B) peroxidase activity were measured in the 1st, 3rd, and 5th scales following 0, 2, and 4 d of heat treatment at 33 °C, 98% RH. Data are means (±SE) for five repeated experiments. Different letters indicate significant differences between treatment times at each scale position according to ANOVA test (*P* < 0.05).

Enzymatic browning of outer scales has been reported to be caused by the autoxidation of QGs, after their deglucosylation (Takahama and Oniki, 2000). Since heating of the outer scale induced its browning, we explored a possible association with changes in QG levels. QG content was determined in the 1st, 3rd, and the 5th scales following 0, 2, and 4 d of heat treatment (33 °C, 98% RH). QG concentrations decreased gradually from the outer scale toward the inner scales on a DW basis (Fig. 4A). The heat treatment induced increasingly high concentrations of QGs in each scale position (Fig. 4A). A similar profile was obtained for POD activity, which was highest in the 1st scale and gradually decreased toward the 3rd and 5th scales (Fig. 4B). Heat treatment for 2 and 4 d induced an increase in POD activity in all the scales examined.

### Transcriptome analysis of outer versus inner scales following heat treatment

To determine the differential transcriptomic response of the 1st and 5th scales to heat treatment, comparative analysis was conducted. Twelve selected pools of mRNA samples, representing the outer (1st) and inner (5th) scales of the onion bulb at three time-points, 0, 24, and 48 h of heat treatment, served for the construction of high-throughput parallel RNA-Seq libraries (Supplementary Table S1 at *JXB* online). Each of the cDNA libraries yielded 16–21 million 100-bp single-end reads. About 85% of the cleaned reads could be mapped to the *de novo* transcript catalog of onion (Galsurker *et al.*, 2017), consisting 45892 contigs with an *N*_50_ value of 1694, median contig length of 965 bp, and mean contig length of 1208.62 bp. This mapping percentage suggested a satisfactory result as compared to the full-length RNA transcript of onion using long-read technology (Sohn *et al.*, 2016).

**Fig. 5.**
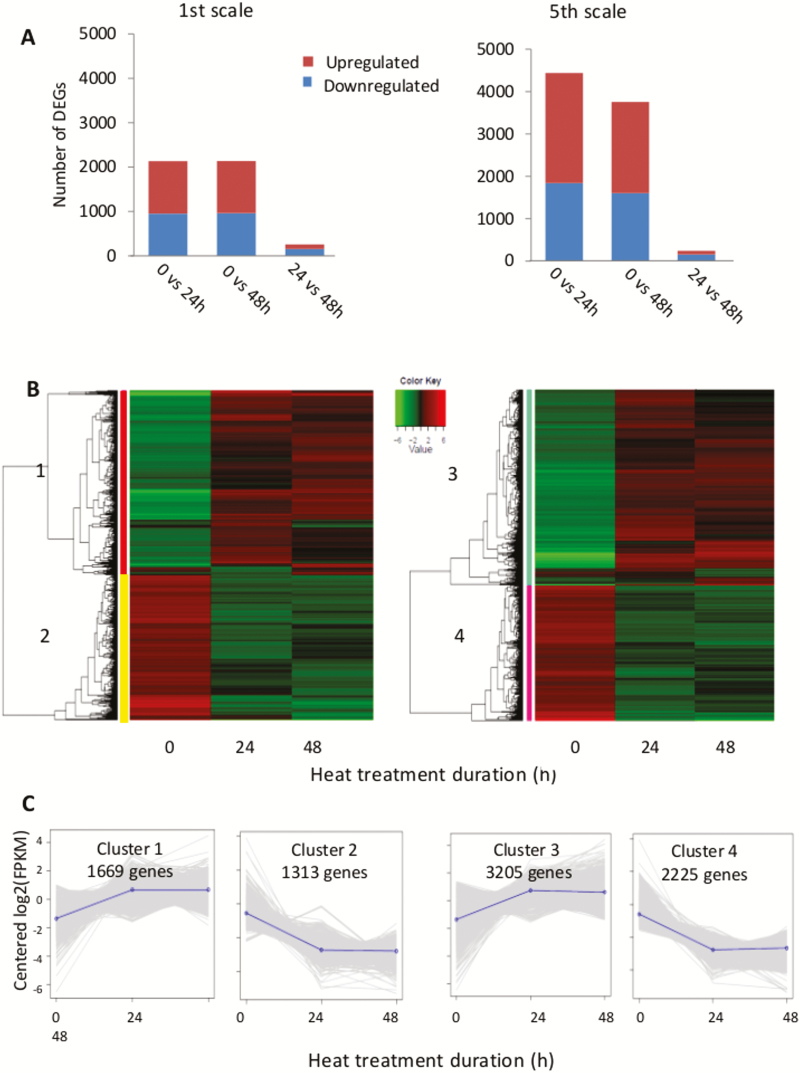
Analysis of the differentially expressed genes (DEGs) and hierarchal cluster analysis of the 1st and 5th onion scales following 0, 24, and 48 h of heat treatment at 33 °C, 98% RH. (A) Total number of DEGs in the pairwise comparisons of three time-points in the 1st and in 5th scales. (B) Heat-map and hierarchical cluster analysis of the 2982 DEGs in the 1st scale (left) and 5430 DEGs in the 5th scale (right). The two main clusters in each scale are represented in red (1) and yellow (2) for the 1st scale, and in green (3) and pink (4) for the 5th scale. (C) Expression of genes in each cluster highlight in (B). The normalized expression value was centered and log_2_-transformed for visualization purposes using a script taken from the Trinity platform (see Methods).

Differentially expressed genes (DEGs) during heat treatment in the 1st and 5th scales were identified by three pairwise comparisons at the three time-points within each scale. We identified a total 2982 DEGs in the 1st scale and 5430 DEGs in the 5th scale during 48 h of heat treatment with FDR<0.001 and a fold-change of either >2 or <–2 (Fig. 5). In general, the number of DEGs in the pairwise comparisons of the three time-points was much higher in the 5th than in the 1st scale (Fig. 5A). In both scales, there were high proportions of DEGs between the 0 h and 24 h time-points and between the 0 h and 48 h time-points, and the lowest proportion was found between the 24 h and 48 h time-points. Thus, most of the changes in gene expression had already occurred after 24 h of treatment and remained constant thereafter. The expression patterns of the total DEGs in the 1st and 5th scales during the heat treatment were studied using two separate hierarchical cluster analyses in at least one pairwise comparison. In the 1st scale, there were two significant clusters of co-expressed genes: cluster 1 containing 1669 up-regulated genes and cluster 2 containing 1313 down-regulated genes (Fig. 5B, C). A similar profile was obtained for the 5th scale, also consisting of two significant clusters: cluster 3 and cluster 4 that contained 3205 up-regulated and 2225 down-regulated genes, respectively (Fig. 5B, C). As no significant changes were found between 24 and 48 h, further analysis focused on the differences between 0 and 24 h of heat treatment.

**Fig. 6.**
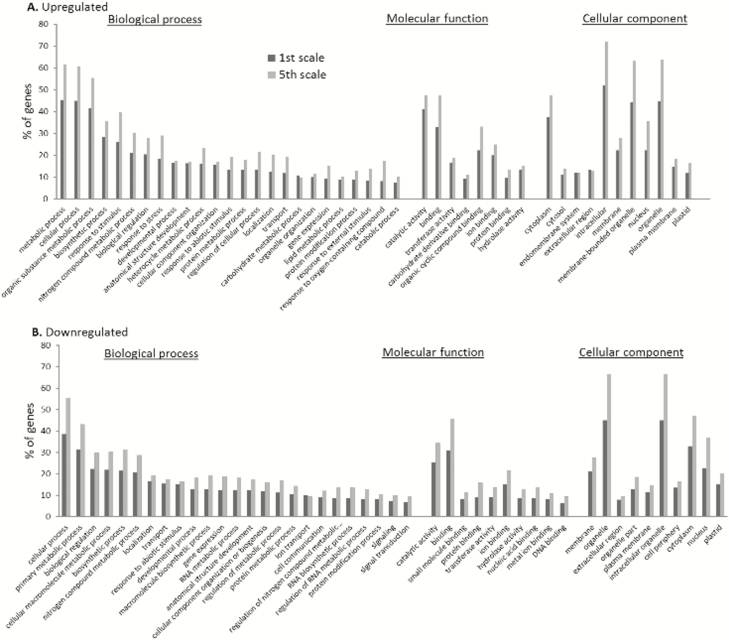
Gene ontology (GO) functional classifications of differentially expressed genes (DEGs) in the 1st and 5th onion scales following heat treatment at 33 °C, 98% RH. Percentage of genes for cellular component, biological process, and molecular function classifications of DEGs in the 1st and 5th scales that were (A) up-regulated or (B) down-regulated following heat treatment.

GO functional classifications of the DEGs in the scales during the heat treatment were analysed. Over-representations of GO terms in the 1st and 5th scales were evaluated to examine which biological processes, molecular functions, and cellular components were mostly influenced by the heat treatment. Most of the heat-induced DEGs represented cellular components associated with intracellular processes, the membrane, and the cytoplasm in both the 1st and 5th scales (Fig. 6A). The biological processes in the set of heat-induced DEGs in both of the scales were cellular processes, organic-substance metabolic processes, and biosynthetic processes. Other over-represented categories of biological processes included nitrogen compound metabolic processes and responses to stimulus. The GO terms for molecular functions that were up-regulated in both of the scales in response to heat included mainly genes encoding proteins with catalytic, binding, or transferase activity. In the cellular component category, the most heat-repressed DEGs in both of the scales were categorized as intracellular organelle, cytoplasm, and nucleus (Fig. 6B). The most abundant biological process categories that were significantly down-regulated in both of the scales in response to heat included cellular processes and biological regulation. Among the notable down-regulated molecular functions in the scales were GO terms related to catalytic activity and binding (Fig. 6B). Overall, the 5th scale showed a similar profile of GO classification distribution compared to the 1st scale, but with a higher number of DEGs in all up- and down-regulated GO categories.

### Common and distinct heat-induced DEGs between outer and inner scales

**Fig. 7.**
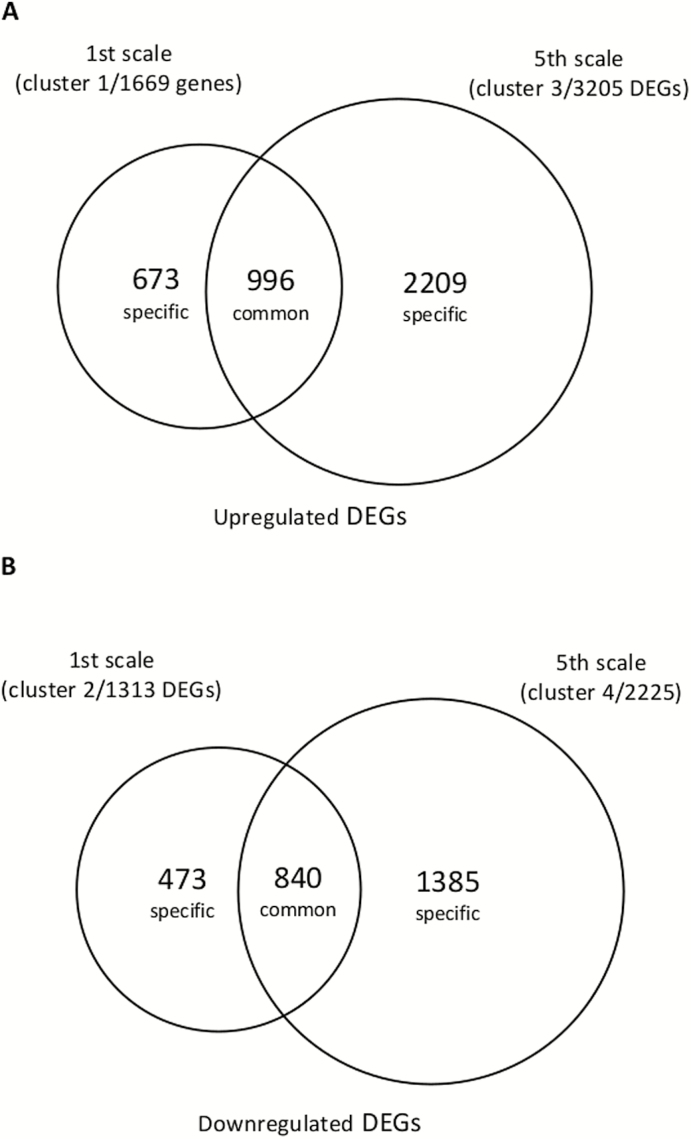
Venn diagram indicating the number differentially expressed genes (DEGs) in the 1st and 5th onion scales after 0, 24, and 48 h of heat treatment at 33 °C, 98% RH. (A) Up-regulated DEGs in clusters 1 and 3 (see Fig. 5). (B) Down-regulated DEGs in clusters 2 and 4. Only genes with an increase or decrease of 2-fold (log_2_) are included.

To examine the unique and shared differential gene expression between the 1st and 5th scales following the heat treatment, a Venn diagram based on cluster comparisons between the scales was constructed. There was partial similarity between the scales; however, high numbers of specific DEGs were also found in each scale (Fig. 7). Of the 1669 and 3205 DEGs that were up-regulated in the 1st and 5th scales, respectively, there was an overlap of 996 that were common to both, whereas 673 and 2209 genes were specifically up-regulated in the 1st and 5th scales, respectively (Fig. 7A). A similar profile was obtained for the comparison of down-regulated DEGs. This showed 473 DEGs specifically down-regulated in the 1st scale in response to heat, 1385 DEGs specifically down-regulated in the 5th scale, and 840 genes that were down-regulated in both (Fig. 7B).

**Fig. 8.**
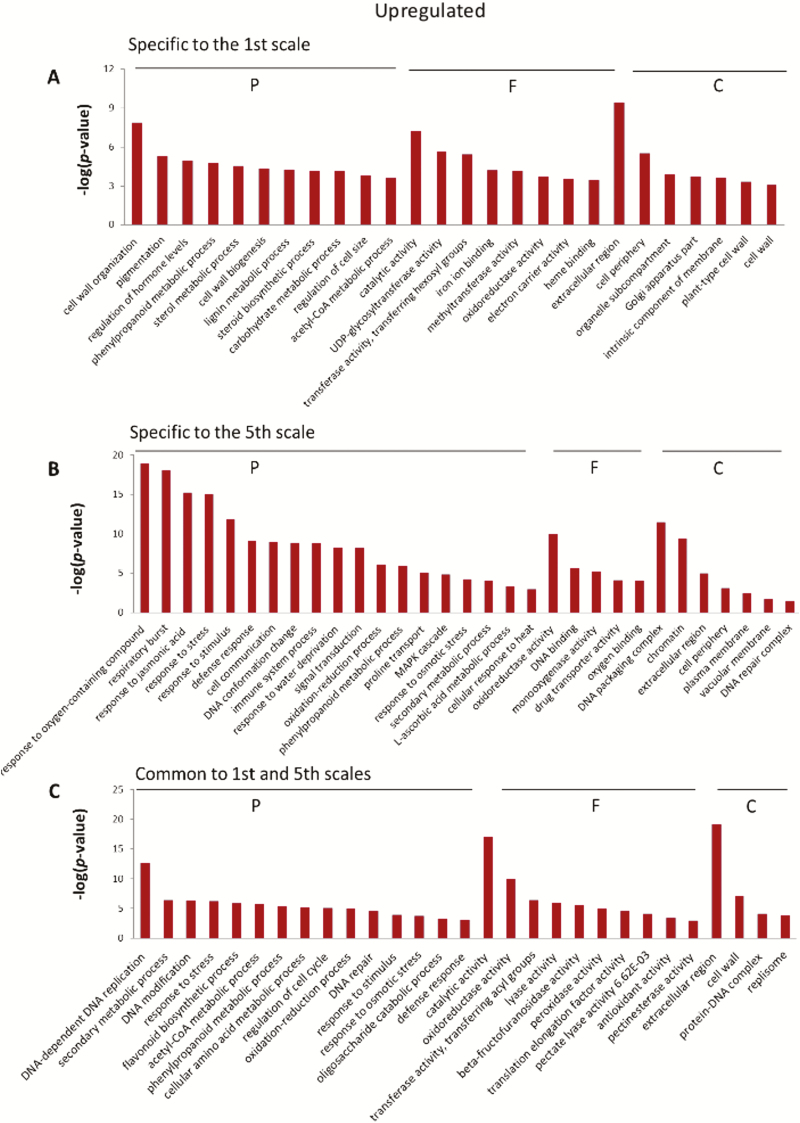

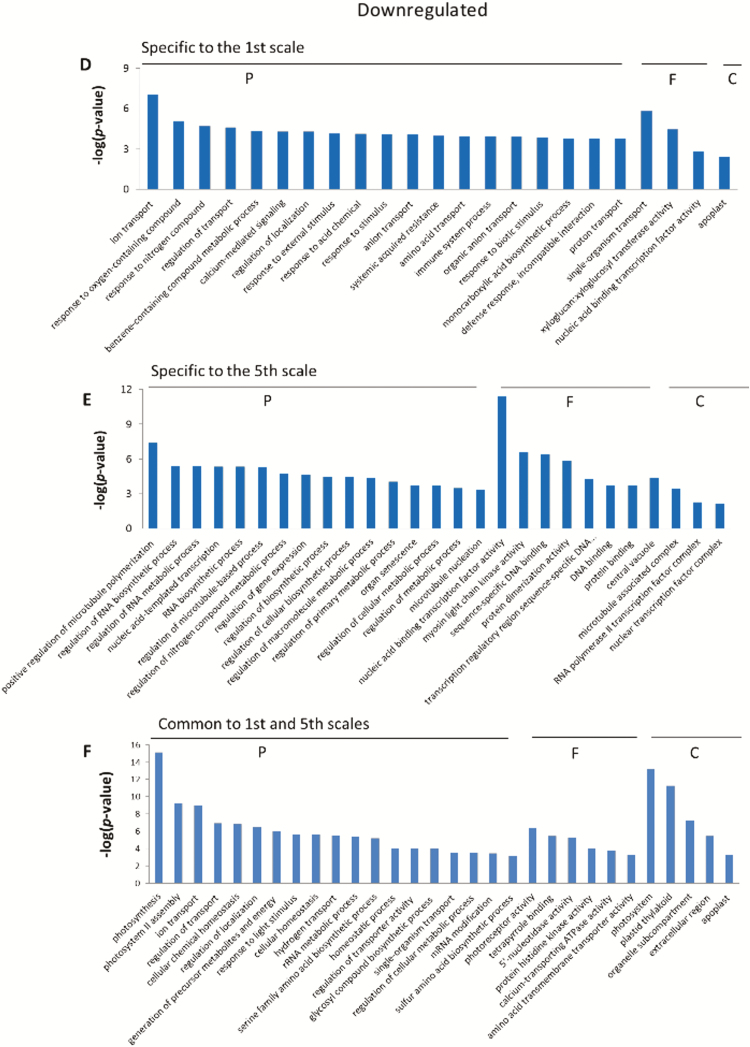
Gene ontology (GO)-enrichment analysis for the specific and common up- and down-regulated differentially expressed genes (DEGs) in Fig. 7. Enrichment of up-regulated genes specific to (A) the 1st scale and (B) the 5th scale. (C) Enrichment of up-regulated genes common to the 1st and 5th scales. Enrichment of down-regulated genes specific to (D) the 1st scale and (E) the 5th scale. (F) Enrichment of down-regulated genes common to the 1st and 5th scales. The groups were subjected to GO-enrichment calculation using Fisher’s Exact Test. The *x*-axes list the most significant GO terms; the *y*-axes represent the negative log of the *P*-values. The GO terms are divided into three categories: cellular components (C), molecular functions (F), and biological process (P).

The specific and common groups of up- and down-regulated DEGs identified for the two different scales were subjected to GO-enrichment analysis using the Blast2GO tool (Fig. 8). Different GO-enrichment profiles were obtained for the groups, revealing the differential effects of the heat treatment on the two scales.

### Defense responses of inner scales following heat treatment

**Fig. 9.**
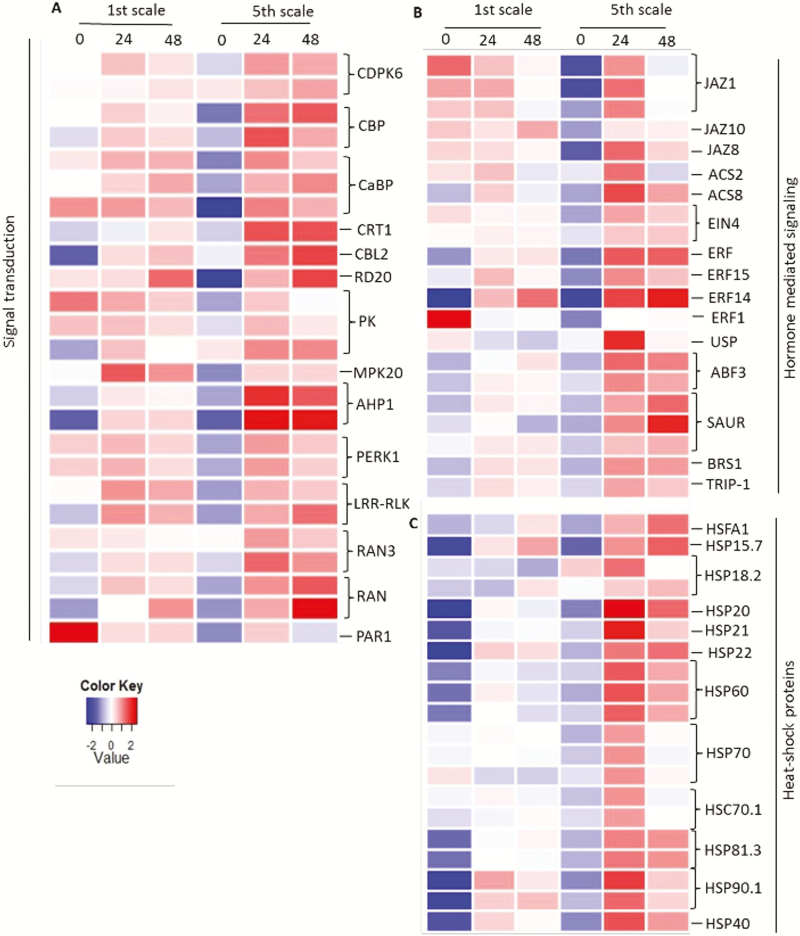

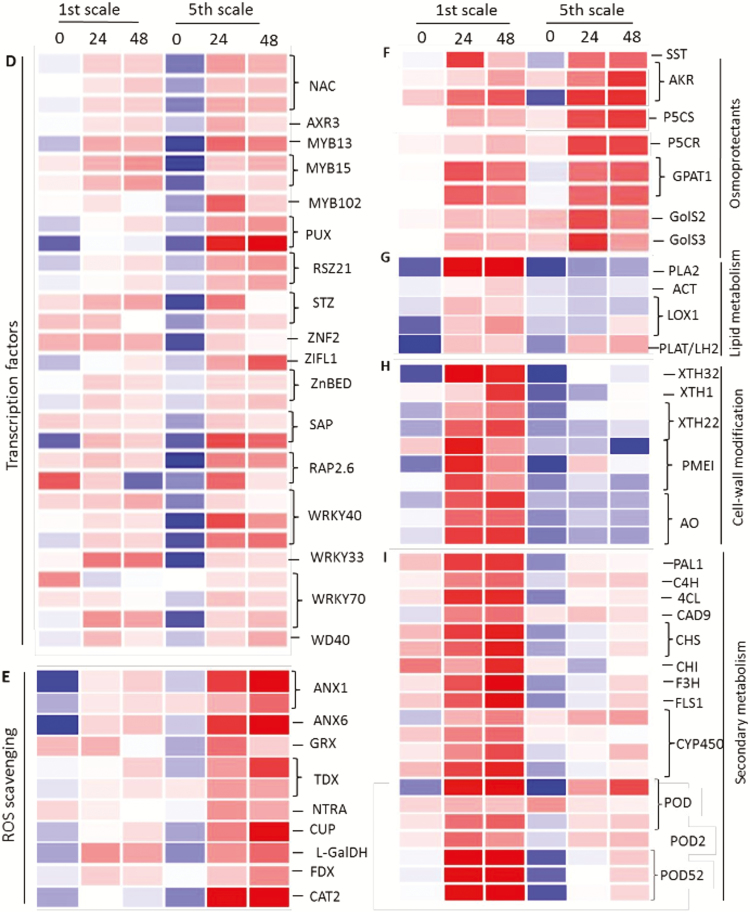
Heat-maps describing the expression profiles of genes related to heat response and browning in the 1st (outer) onion scale compared to the 5th (inner) scale. Genes related to (A) signal transduction, (B) hormone-mediating signaling, (C) heat-shock proteins, (D) transcription factors, (E) ROS scavenging, (F) osmoprotectant metabolism, (G) lipid metabolism, (H) cell-wall modification, and (I) secondary metabolism. Measurements were performed at 0, 24, and 48 h of heat treatment at 33 °C, 98% RH. Full gene names are provided in Supplementary Table S2.

GO enrichment analysis emphasized the differential responses of the 1st (outer) and 5th (inner) scales to the applied heat treatment. In contrast to the 1st scale, the 5th managed to survive the heat treatment. Therefore, we examined whether this was reflected in differential expression of genes involved in heat resistance. We focused our analysis on the expression of abiotic stress defense-responsive genes that are expected to be involved in heat resistance. The genes associated with defense response and heat tolerance were subdivided into several pathways, such as signaling, stress-related hormones, transcriptional regulation, stress-responsive genes encoding heat-shock proteins (HSPs), scavenging of reactive oxygen species (ROS), and osmoprotection metabolism. In general, most of these genes were induced by the heat treatment in both of the scales, although the enhancement in the 5th scale was much more pronounced, resulting in higher expression levels (Fig. 9; Supplementary Table S2).

Genes related to the signaling pathway associated with stress responses were more highly expressed in the 5th scale under the heat treatment, which started with low expression at time 0 h. Most of these genes were dramatically up-regulated after 24 or 48 h of heat treatment. In contrast, in the 1st scale most of the same group of genes were only slightly enhanced during the heat treatment (Fig. 9A). The sensing of abiotic stress triggers several signaling pathways in plants, including those of calcium-dependent protein kinases (CDPKs) and calcium/calmodulin-dependent protein kinases (CCaMKs). Signaling-related genes such as those encoding CDPK6, calmodulin-binding protein (CBP), and calcium-binding protein (CaBP) were greatly up-regulated in the 5th scale compared to the 1st during the heat treatment. Other signaling-related genes had high expression profiles in the 5th scale, especially during heat treatment, such as those encoding mitogen-activated kinase (MAPK20), calcineurin B-like 2 (CBL2), histidine-containing phosphotransmitter 1 (AHP1), and Ras-related nuclear protein (RAN) (Fig. 9A).

Genes involved in stress-related hormone-signaling pathways were dramatically up-regulated under the heat treatment in the 5th scale compared to the 1st (Fig. 9B). Ethylene-responsive genes, especially those whose products are ethylene-responsive factors (ERFs), and genes associated with ethylene biosynthesis, such as 1-aminocyclopropane-1-carboxylate synthases (ACS2 and ACS8), showed higher expression in the 5th scale, mainly at 24 h after the heat treatment. Other hormone-related genes that showed high expression in the 5th scale compared to the 1st during the heat treatment were the abscisic acid (ABA)-responsive gene that encodes the ABA-responsive elements-binding factor 3 (ABF3) and auxin-responsive genes, such as small auxin-up RNA (SAUR) genes (Fig. 9B).

Activation of various plant responses is mediated in many cases by transcriptional regulation of defense-related genes. Analysis of transcription factor (TF)-encoding genes revealed higher expression of genes encoding MYB-domain family proteins (MYB13, MYB15, and MYB102), WRKY DNA-binding proteins (WRKY33, WRKY40, and WRKY70), NAC-domain family (NAC), and UBX domain-containing family (PUX) in the 5th scale after 48 h of heat treatment (Fig. 9D). More moderate changes in the expression of these genes were observed in the 1st scale compared to the 5th.

Examination of the changes in expression of genes encoding HSPs such as Hsp20, Hsp40, Hsp60, Hsp70, and Hsp90 revealed greater up-regulation in the 5th scale, most of them after 24 h of heat treatment, compared to a more moderate increase in the 1st scale (Fig. 9C).

Analysis of genes related to ROS scavenging- involved in the response to antioxidative stress showed more significant up-regulation in the 5th scale compared to the 1st after the heat treatment, highlighting the importance of ROS-scavenging genes in the defense response. These redox state-related genes encode the annexin family proteins (ANX1 and ANX6), glutaredoxin (GRX), thioredoxin (TDX), NADPH-dependent TDX reductase A (NTRA), cupredoxin (CUP), ferredoxin (FDX), and catalase (CAT2) (Fig. 9E).

Examination of the DEGs related to synthesis of osmoprotectant solutes generally showed up-regulation and high expression following the heat treatment. This was especially the case in the 5th scale, whereas induction in the 1st scale was more moderate. For example, expression of genes encoding sucrose:sucrose 1-fructosyltransferase (SST), pyrroline-5-carboxylate synthase (P5CS), and P5C reductase (P5CR) was strongly induced in the 5th scale compared to the 1st .

### Expression of genes related to tissue browning in the outer scale

We examined the differential expression of genes related to pathways that could be involved in scale browning following heat treatment, such as those associated with secondary metabolism, lipid metabolism, and cell-wall modifications. Most of the DEGs were involved in phenylpropanoid and flavonoid metabolism, especially in the 1st scale (Fig. 9I). Among them were genes encoding phenylalanine ammonia-lyase 1 (PAL1), cinnamate-4-hydroxylase (C4H), flavanone 3-hydroxylase (F3H), chalcone synthase (CHS), cytochrome P450 (CYP450), and POD. Several genes involved in cell-wall loosening and modification, such as those encoding xyloglucan endotransglucosylase 1 (XTH1), pectin methylesterase inhibitor (PMEI), and L-ascorbate oxidase (AO), were induced in the 1st scale during the heat treatment, and were more highly expressed in the 1st scale than in the 5th (Fig. 9H). With respect to lipid metabolism, genes encoding key enzymes involved in lipid-degradation pathways were identified as being more highly expressed after the heat treatment in the 1st scale compared to the 5th. Phospholipase A2 (PLA2) family proteins, acyl-CoA thioesterase (ACT) family proteins, and lipoxygenase 1 (LOX1) were among the more highly up-regulated genes in the 1st scale compared with the 5th (Fig. 9G).

## Discussion

### Inner onion scales demonstrate higher tolerance to heat treatment

Skin formation of onion bulb involves scale desiccation accompanied by senescence, resulting in PCD and browning of the outer scales (Galsurker *et al.*, 2017). These processes can be induced by post-harvest curing of bulbs, using heat treatment and high humidity to dry out the outer scales and induce them to develop brown skins (Downes *et al.*, 2009; Chope *et al.*, 2012; Eshel *et al.*, 2014). Here, we have demonstrated that differential biological responses of onion bulb scales occur in response to heat treatment depending on their position in the bulb. We have shown that the ability to form a dry brown skin during heat treatment is unique to the outer scales. Significant water loss or browning was not detected in exposed inner scales, even after an extended heat treatment, suggesting resistance of these scales to the heat stress, which is probably based on biological processes activated by the stress (Fig. 1). Although the initial dry matter content was similar in all scales (not shown), the decrease in RWC over 22 d of heating was found only in the outer scale, suggesting that water loss was not affected by dry matter content, as shown in higher temperature treatments (Sharma *et al.*, 2015).

**Fig. 10.**
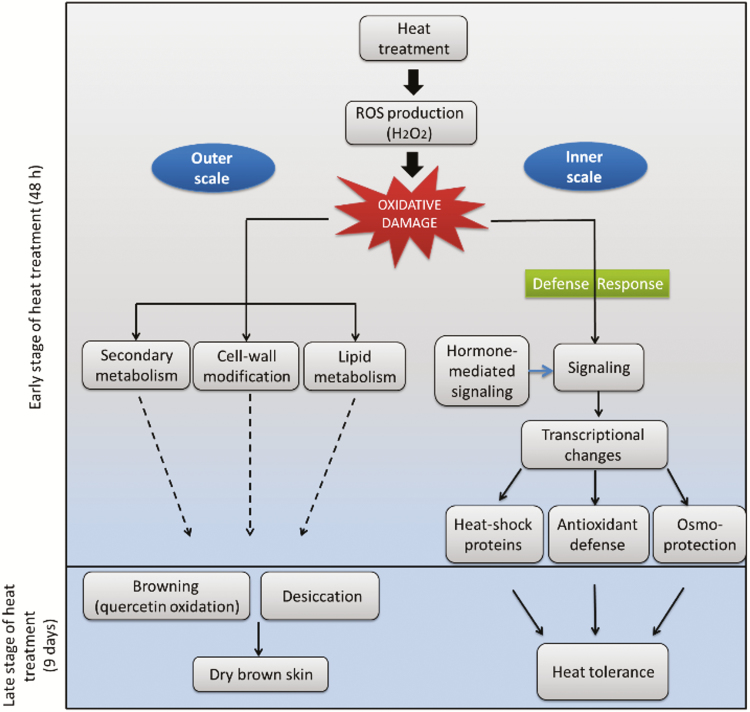
Proposed model for the response of onion scales to heat treatment. The model is based on the over-representation of the corresponding genes in the 1st (outer) and 5th (inner) scales.

Comparative transcriptomic analysis revealed differential responses of the outer and inner scales to the heat treatment. A higher number of genes were differentially expressed in the inner compared to outer scales during the heat treatment, suggesting a stress-resistance response in the inner scales (Fig. 5). The most over-represented metabolic processes in the inner scales were associated with various stress and defense responses (Fig. 8B). This suggests that the inner scales respond to the heat stress by activating defense-response mechanisms. An induced heat resistance of this kind is expected to employ pathways such as signal transduction, hormone-mediated signaling, HSP activation, action of TFs, ROS-scavenging activities, and osmoprotectant metabolism, which were apparent in the functions of the over-represented genes in the transcriptome (Fig. 9). We propose a hypothetical model for the differential heat responses of the outer versus inner scales (Fig. 10). Based on the observed structural, physiological, and biochemical changes of the different scales and the changes in their transcriptomes following the heat treatment, we suggest that defense responses are significantly activated by heat in the inner scales, generating tolerance, whereas processes related to rapid senescence and skin formation occur in the outer scale.

### Signal-transduction genes

Activation of abiotic stress responses in plants is mediated by signal-transduction pathways, which transduce the initial stress signal to activate gene expression and defense mechanisms (reviewed by Huang *et al.*, 2012). The ability of inner scales to remain viable probably also depends on signal transduction and defense responses. The transcriptome analysis revealed over-representation of genes associated with signal transduction in the inner scales, which fits with maintenance of viability. Enrichment of genes involved in calcium/calmodulin-signaling pathways including genes encoding for CDPK, CBP, CaBP, and CBL2 in the inner scale under the heat treatment (Fig. 9A) could be related to the known role of calcium and calmodulin in heat-stress signaling in plants (Liu *et al.*, 2003; Zhang *et al.*, 2009). Other genes encoding MAPK20 and RAN proteins were also induced in the inner scales under the heat treatment (Fig. 9A). MAPK cascades play an important role in signal-transduction pathways in plants and function ubiquitously in many responses to external signals (Gupta and Kaur, 2005). RAN is known to be involved in mediating responses to abiotic stresses such as heat, salt, and drought stresses (Ferreira *et al.*, 2006; Yoshimura *et al.*, 2008; Zang *et al.*, 2010).

### Hormone-signaling genes

Signaling pathways involving plant hormones have been proposed to play an important role in plant heat tolerance (Kotak *et al.*, 2007). ROS are involved as second messengers in plant hormone-signaling cascades that play an essential role in mediating plant stress responses (Bartoli *et al.*, 2013). Genes involved in hormone-signaling pathways were induced in response to heat treatment of onion scales (Fig. 9B). Early auxin-responsive genes (encoding SAUR) and ethylene-related signaling genes, including genes encoding ERFs, ethylene insensitive (EIN), and ABF3, were over-represented in the inner scales after heat treatment (Fig. 9B). Ethylene is well known to be involved with the response to abiotic stresses (van Loon *et al.*, 2006; Grennan, 2008), and ERFs and ABF3 are known to play an important role in plant stress responses (Choi *et al.*, 2013).

### Transcription factors

Various TF genes were differentially over-represented in the inner scales after heat treatment, mainly encoding NAC-domain family proteins, WRKY DNA-binding proteins, MYB-domain family proteins, and WD-40 repeat protein families (Fig. 9D). WRKY TF genes are mainly involved in the regulation of defense against biotic and abiotic stresses (Ülker *et al.*, 2007; Rushton *et al.*, 2010; Chen *et al.*, 2012; Li *et al.*, 2013; Bakshi and Oelmüller, 2014) including regulation of heat tolerance (Dang *et al.*, 2013). WD-40 repeat proteins play critical roles in specific developmental events, as well as in plant tolerance to abiotic stresses (Mishra *et al.*, 2012).

### Genes related to heat-shock proteins

Various genes related to HSPs were over-represented in the inner scales under heat treatment, among them heat-shock factor A1 (HSFA1), and genes encoding HSP40, HSP60, HSP70, HSP90, and HSP101 (Fig. 9C). HSFA1 has been shown to play a role in the response to heat and other stresses in Arabidopsis (Liu *et al.*, 2011). HSPs, including HSP60, HSP70, HSP90, and small HSPs, have critical roles in regulating protein quality by renaturing proteins denatured by heat stress (Ohama *et al.*, 2017). In Arabidopsis, HSP101 is involved in increasing chloroplast thermotolerance during heat stress (Myouga *et al.*, 2006). Plants synthesize HSPs in response to high temperature to act as molecular chaperones that prevent denaturation, to assist in refolding damaged proteins, and to maintain cellular homeostasis under high temperature (Wang *et al.*, 2004; Ouyang *et al.*, 2007).

### Genes associated with ROS scavenging

Several genes encoding ROS scavengers were also highly expressed in the inner scales after heat treatment, including members of large families such as *TRX*, *GRX*, *ANX*, *FDX*, and *CAT2* (Fig. 9E). ROS production and accumulation are known to be involved with heat stress, causing oxidation of cellular components and loss of organelle integrity (Mittler, 2002; Almeselmani *et al.*, 2006). Toxic ROS produced by heat stress may be scavenged mainly by enzymatic detoxification systems. Up-regulation of ROS-scavenging enzymes such as ascorbate peroxidase (Davletova *et al.*, 2005; Locato *et al.*, 2008), CAT (Locato *et al.*, 2008), and glutathione-S-transferase (Zhao *et al.*, 2009) has been reported to be involved in heat tolerance. Moreover, oxidized products have been found to be enzymatically neutralized by GRX and TRX (Davies, 2005). In Arabidopsis, induction of TRX results in thermotolerance (Lee *et al.*, 2009), and expression of plant FDX-like protein enhances tolerance to heat stress (Lin *et al.*, 2015). Thus, induction of genes encoding for such ROS scavengers could be part of the mechanisms that enable the inner scales of onion to withstand heat stress.

### Genes related to osmoprotectant metabolism

Our hypothetical model also suggests that induced synthesis/accumulation of compatible solutes (osmolytes) provides a mechanism that has a role in heat-stress tolerance in the inner scales (Fig. 10). Genes encoding enzymes involved in the synthesis of osmoprotectants and carbohydrate metabolism were up-regulated in the inner scales (Fig. 9F). The fructan biosynthesis- related gene *SST* was over-represented in the inner scales following heating. Fructans are polymers of fructose that serve as an important storage carbohydrate in onion (Pollock and Cairns, 1991), and may have a role in abiotic stress tolerance (Hendry and Wallace, 1993; Spollen and Nelson, 1994). Transgenic rice and tobacco expressing wheat *1-SST* or *6-SFT* genes accumulated more fructans and showed higher freezing tolerance (Li *et al.*, 2007; Kawakami *et al.*, 2008). Other genes encoding the galactinol synthase family (GolS) were induced in the inner scale under the heat treatment (Fig. 9F). Busch *et al.* (2005) suggested that heat stress induces the production of specific sugars, such as galactinol by galactinol synthases, and raffinose is involved in the Arabidopsis heat-stress response. It is possible that in the onion system enhanced accumulation of fructans plays a role in the heat tolerance of the inner scales.

Over-representation of genes encoding P5CS, a key enzyme in proline biosynthesis, and P5C1, which further reduces pyrroline-5-carboxylate to proline, was found in the inner scales under heat treatment (Fig. 9F). Proline synthesis is activated during stress conditions and its degradation is enhanced during stress recovery (Mattioli *et al.*, 2009; Rai and Penna, 2013). Overexpression of the enzyme P5CS in transgenic plants of tobacco and petunia under drought led to the accumulation and increased cellular levels of proline, which acted as an osmoprotectant, thereby inducing drought tolerance (Kishor *et al.*, 1995; Yamada *et al.*, 2005). In addition, it has been proposed that the accumulation of osmolytes confers protection against oxidative damage that impairs normal cell function (Bartels and Sunkar, 2005; Mittler, 2006). Thus, induction of genes related to proline metabolism in the inner scales supports a role of proline in the observed resistance of these scales to heat stress.

### Browning is unique to the outer scale due to specific enzymatic responses

Following heat treatment, liquid suspensions of the powdered outer scale, but not of inner scales, could develop brown color under heated incubation (Fig. 3A). Browning of the outer scale is probably driven by enzymatic activity, as indicated by its sensitivity to boiling (Fig. 3B). The browning process requires direct interaction of phenolic compounds with the relevant enzymes, which may be mediated by loss of cell compartmentalization in the senescing cells of the outer scale. These results are in agreement with a previous study that demonstrated that onion-scale browning is an enzymatic process, associated with POD-dependent oxidation of phenolic compounds (Takahama, 2004). The decrease in the skin *H* value after the curing of brown onion cultivars at 28 °C has been linked to decreases in concentrations of individual flavonols, possibly due to their oxidation into brown pigmented compounds at higher temperatures (Cools *et al.*, 2010).

We found a correlation between outer-scale browning and elevated ion leakage, indicating the development of membrane damage during the heat treatment (Fig. 2A). Disrupted membrane integrity of the cells of the outer scale may enable the release of enzymes that lead to oxidation and deglucosidation of the phenolic compound QG. The quercetin that is formed can be further oxidized by POD, resulting in the formation of multiple brown-colored pigments that contribute to onion-skin browning (Cools *et al.*, 2010). Browning components (i.e. QG and POD) showed a gradual increase from the inner to outer scales (Fig. 4), suggesting that their high levels in the outer scale are involved in tissue-browning. Our results are in agreement with a previous study that found that QG concentration and POD activity decrease from the outer to inner bulb scales (Hirota *et al.*, 1998). The decrease in QG concentration in the inner scales is derived from the structure of these scales, which contain abaxial epidermal tissue with a thick mesophyll cell tissue layer beneath; hence the decrease was suggested to be caused by dilution of QGs, which are present only in the epidermal tissue and not the mesophyll (Trammell and Peterson, 1976). The differential levels of QGs and POD in the various onion scales, together with the membrane damage to the scale cells, are associated with browning of only the outer scale.

Transcriptomic results revealed the involvement of specific biological processes and regulatory mechanisms in determining outer-scale browning. Most over-represented metabolic genes in the outer scale during the heat treatment were associated with cell-wall organization, pigmentation, and phenylpropanoid biosynthesis (Fig. 8A), and these pathways are expected to be involved in browning and skin formation in the outer scale (Fig. 10). Genes involved in secondary metabolism, mainly in the phenylpropanoid- and flavonoid-biosynthesis pathways, were highly induced in the outer scale under heat treatment (Fig. 9I), suggesting a shift from primary to secondary metabolism. They included genes encoding PAL1, C4H, 4CL, CHS, and F3H. PAL is the first dedicated enzyme in the phenylpropanoid pathway, converting phenylalanine into trans-cinnamic acid and providing a substrate for further synthesis of phenolic compounds (Hahlbrock and Scheel, 1989). Onion scales contain QGs (quercetin 4’-glucoside and quercetin 3,4’-diglucoside) as major phenolics, accounting for 80% of all flavonoids (Hirota *et al.*, 1998; Slimestad *et al.*, 2007). The over-representation of QG synthesis genes was positively correlated with the accumulation of QGs in the outer scale.

Genes encoding POD and CYP450 were also highly represented in the outer scales in response to heat treatment (Fig. 9I). CYP450 is a large plant protein family that might contribute to extensive modifications of various secondary compounds. It plays an important role in flavone biosynthesis and is expressed during tissue browning (Wang *et al.*, 2013).

Since the outer scale is in a process of senescence, it is therefore more prone to oxidative stress, which can cause lipid peroxidation and cell-wall degradation that leads to loss of compartmentalization. Genes encoding key enzymes of lipid-degradation pathways, such as PLA2 and LOX1, which are critical in lipid peroxidation, were up-regulated in the outer compared to the inner scales under heat treatment (Fig. 9G). Enrichment of genes associated with cell-wall degradation were found in the outer scale, including XTH, PMEI, and AO (Fig. 9H). These genes encode for apoplastic enzymes linked to cell-wall modification, regulation of stress perception, and signal transduction (Pignocchi and Foyer, 2003). Indeed, our transcriptomic results support the hypothesis that loss of compartmentalization causes the release of the phenolic compounds—QGs—and the enzyme that catalyses their deglucosidation, resulting in the formation of quercetin, which can then be autoxidized by POD to form the brown-colored pigments in the skin (Takahama, 2004).

## Conclusions

The transcriptome analysis in this study led to a putative model for differential heat responses of the outer and inner scales. The model suggests that the inner scales express heat tolerance, as compared to the heat sensitivity of the outer scale that results in browning and skin development (Fig. 10). This differential response can be explained by the different physiological ages of the scales, with older ones on the outside and younger ones toward the inside (Brewster, 2008; Galsurker *et al.*, 2017). The different biological processes suggested to occur by our study in the inner and outer scales are probably related to their different functions. Outer skins are required to protect the bulb against disease by providing both physical and biochemical barriers to infection by pathogens (Eshel *et al.*, 2014; Mishra *et al.*, 2014).

The identification of genes involved in heat-tolerance mechanisms in the inner scales and browning development in the outer scale lays the ground for additional proteomic and metabolomic screens to further understand onion-skin formation. It also represents a valuable resource for the identification of candidate heat-tolerance genes, thus providing important information on the mechanisms underlying heat tolerance.

## Supplementary data

Supplementary data are available at *JXB* online.

Table S1. Overview of the RNA-Seq data obtained from the different onion scales during heat treatment.

Table S2. Gene abbreviations and their full names, listed according to the functional groups in Fig. 9.

Supplementary Tables S1-S2
