# Supplementary material for: Differential response to heat stress in outer and inner onion bulb scales
Source: J Exp Bot. 2018 May 18;69(16):4047–64. doi: 10.1093/jxb/ery189 (PMC6054243; doi:10.1093/jxb/ery189)
Supplement: Supplementary Tables S1-S2 [file ery189_suppl_supplementary_tables_s1-s2.pdf]

Differential response to heat stress in outer and inner onion bulb scales

Ortal Galsurker[a,b], Adi Doron-Faigenboim[c], Paula Teper-Bamnolker[a], Avinoam Daus[a], Amnon Lers[a] and Dani Eshel[a*]

Table S1. Overview of the RNA-Seq data obtained from the different onion scales during heat treatment.

Scale position†	Heat treatment (h)	No. clean reads	No. mapping reads	% mapping
1st (a)	0	20,962,091	17,926,086	85.5
1st (b)	0	17,903,262	14,995,844	83.8
5th (a)	0	17,637,477	14,698,083	83.3
5th (b)	0	16,373,596	13,699,655	83.7
1st (a)	24	20,434,805	17,195,861	84.1
1st (b)	24	21,032,654	17,863,251	84.9
5th (a)	24	18,702,894	15,771,148	84.3
5th (b)	24	23,647,493	19,884,576	84.1
1st (a)	48	19,559,693	16,497,463	84.3
1st (b)	48	20,022,292	16,932,439	84.6
5th (a)	48	19,741,407	16,667,334	84.4
5th (b)	48	19,913,319	16,771,331	84.2

†(a) and (b) represent two replicates.

Table S2. Gene abbreviations and their full names; Presented according to the functional groups at Fig. 9.

A Signal transduction

CDPK6	Calcium-dependent protein kinase 6
CBP	Calmodulin-binding protein
CaBP	Calcium-binding protein
CRT1	Calreticulin 1
CBL2	Calcineurin B-like 2
RD20	Responsive to dessication 20
PK	Protein kinase
MPK20	Mitogen-activated kinase 20
AHP1	Histidine-containing phosphotransmitter 1
PERK1	Proline extensin-like receptor kinase 1
LRR-RLK	Leucine-rich repeat receptor-like transmembrane protein kinase
RAN3	Ras-related nuclear protein-3
RAN	Ras-related nuclear protein
PAR1	photoassimilate-responsive protein-related

B Hormone mediated signaling

JAZ1	Jasmonate-zim-domain protein 1
JAZ10	Jasmonate-zim-domain protein 10
JAZ8	Jasmonate-zim-domain protein 8
ACS2	1-aminocyclopropane-1-carboxylate synthase 2
ACS8	1-aminocyclopropane-1-carboxylate synthase 8
EIN4	Ethylene Insensitive 4
ERF	Ethylene-responsive element binding factor
ERF15	Ethylene-responsive element binding factor 15
ERF14	Ethylene-responsive element binding factor 14
ERF1	Ethylene-responsive element binding factor 1
USP	Universal stress protein
ABF3	Abscisic acid responsive elements-binding factor 3
SAUR	Small auxin up RNA
BRS1	BRI1 SUPPRESSOR 1
TRIP1	TGF-beta receptor interacting protein 1

C Heat shock protein

HSFA1	Heat shock factor A 1
HSP15.7	15.7 kDa class I-related small heat shock protein-like
HSP18.2	Heat shock protein 18.2
HSP20	Heat shock protein 20
HSP21	Heat shock protein 21
HSP22	Heat shock protein 22
HSP60	Heat shock protein 60
HSP70	Heat shock protein 70
HSC70.1	heat shock cognate protein 70.1
HSP81.3	Heat shock protein 81.3

HSP90.1	Heat shock protein 90.1
HSP40	Heat shock protein 40

D <u>Transcription factors</u>

NAC	NAC domain transcriptional regulator superfamily protein
AXR3	Auxin resistant 3; transcription factor
MYB13	MYB domain protein 13
MYB15	MYB domain protein 14
MYB102	MYB domain protein 102
PUX	UBX domain-containing protein
RSZ21	RS-containing zinc finger protein 21
STZ	Salt tolerance zinc finger
ZNF2	Zinc-finger protein 2
ZIFL1	Zinc induced facilitator-like 1
ZnBED	BED zinc finger ;hAT family dimerisation domain
SAP	A20/AN1-like zinc finger family protein stress-associated protein
RAP2.6	Related to AP2 6
WRKY40	WRKY DNA-binding protein 40
WRKY33	WRKY DNA-binding protein 33
WRKY70	WRKY DNA-binding protein 70
WD40	Transducin/WD40 repeat-like superfamily protein

E <u>ROS scavenging</u>

ANX1	Annexin 1
ANX6	Annexin 6
GRX	Glutaredoxin-like protein
TDX	Thioredoxin H-type 1
NTRA	NADPH-dependent TDX reductase A
CUP	Cupredoxin superfamily protein
L-GalDH	L-galactose dehydrogenase
FDX	2Fe-2S ferredoxin-like superfamily protein
CAT2	Catalase 2

F <u>Osmoprotectants</u>

SST	Sucrose:sucrose 1-fructosyltransferase
AKR	Aldo/keto reductase family protein
P5CS	Pyrroline-5-carboxylate synthase
P5CR	Pyrroline-5- carboxylate (P5C) reductase
GPAT1	Glycerol-3-phosphate acyltransferase 1
GolS2	Galactinol synthase 2
GolS3	Galactinol synthase 3

G <u>Lipid metabolism</u>

PLA2	Phospholipase A2 family protein
ACT	Acyl-CoA thioesterase family protein
LOX1	Lipoxygenase 1
PLAT/LH2	Lipase/lipooxygenase, PLAT/LH2 family protein

H Cell wall modification

XTH32	xyloglucan endotransglucosylase/hydrolase 32
XTH1	xyloglucan endotransglucosylase/hydrolase 1
XTH22	Xyloglucan endotransglucosylase/hydrolase family protein
PMEI	Pectin methylesterase inhibitor superfamily
AO	L-ascorbate oxidase

I Secondary metabolism

PAL1	PHE ammonia lyase 1
C4H	Cinnamate-4-hydroxylase
4CL	4-coumarate:CoA ligase 3
CAD9	Cinnamyl alcohol dehydrogenase 9
CHS	Chalcone synthase family protein
CHI	Chalcone-flavanone isomerase family protein
F3H	Flavanone 3-hydroxylase
FLS1	Flavonol synthase 1
CYP450	Cytochrome P450 superfamily protein
POD	Peroxidase
POD2	Peroxidase 2
POD52	Peroxidase 52
